# Reversible structural changes in the influenza hemagglutinin precursor at membrane fusion pH

**DOI:** 10.1073/pnas.2208011119

**Published:** 2022-08-08

**Authors:** Eva Garcia-Moro, Jie Zhang, Lesley J. Calder, Nick R. Brown, Steven J. Gamblin, John J. Skehel, Peter B. Rosenthal

**Affiliations:** ^a^Structural Biology of Cells and Viruses Laboratory, Francis Crick Institute, NW1 AT London, United Kingdom;; ^b^Structural Biology of Disease Processes Laboratory, Francis Crick Institute, NW1 AT London, United Kingdom

**Keywords:** influenza, membrane fusion, hemagglutinin, cryo-EM, protein folding

## Abstract

The subunits of the influenza hemagglutinin (HA) trimer are synthesized as single-chain precursors (HA0s) that are proteolytically cleaved into the disulfide-linked polypeptides HA1 and HA2. Cleavage is required for activation of membrane fusion at low pH, which occurs at the beginning of infection following transfer of cell-surface–bound viruses into endosomes. Activation results in extensive changes in the conformation of cleaved HA. To establish the overall contribution of cleavage to the mechanism of HA-mediated membrane fusion, we used cryogenic electron microscopy (cryo-EM) to directly image HA0 at neutral and low pH. We found extensive pH-induced structural changes, some of which were similar to those described for intermediates in the refolding of cleaved HA at low pH. They involve a partial extension of the long central coiled coil formed by melting of the preexisting secondary structure, threading it between the membrane-distal domains, and subsequent refolding as extended helices. The fusion peptide, covalently linked at its N terminus, adopts an amphipathic helical conformation over part of its length and is repositioned and packed against a complementary surface groove of conserved residues. Furthermore, and in contrast to cleaved HA, the changes in HA0 structure at low pH are reversible on reincubation at neutral pH. We discuss the implications of covalently restricted HA0 refolding for the cleaved HA conformational changes that mediate membrane fusion and for the action of antiviral drug candidates and cross-reactive anti-HA antibodies that can block influenza infectivity.

## Significance

Hemagglutinin (HA) is the receptor binding and membrane fusion glycoprotein of influenza virus. Like other virus fusion glycoproteins such as those of HIV and Ebola, HA is synthesized as a precursor (HA0) that requires cleavage for fusion activity and, for influenza, exposure to low pH. Studies by X-ray and cryogenic electron microscopy (cryo-EM) have characterized conformational changes in HA that occur at membrane fusion pH. Here, using cryo-EM, we report that there are extensive changes to the structure of HA0 at low pH but that, unlike the changes in HA, the changes are reversible on return to neutral pH. The low-pH structure of HA0 is considered an indicator of potential intermediates in the conformational changes in HA at fusion pH.

The membranes of lipid enveloped viruses fuse with cellular membranes at the beginning of infection to deliver their genetic material into cells. For some viruses, fusion is at the cell surface; for others, it occurs following transfer of receptor-bound viruses into endosomes. Influenza viruses are in the second group and the virus glycoprotein involved in both receptor-binding and low-pH–triggered membrane fusion is hemagglutinin (HA). HA is synthesized as a precursor, HA0, that is proteolytically cleaved during virus replication into the two disulphide-linked components of infectious virus hemagglutinin, HA1 and HA2 (1–4). For 14 of the 16 HA subtypes, cleavage is by trypsin-like enzymes (5, 6) at an arginine residue that immediately precedes the N terminus of HA2 (7). For some HA0s of the two remaining subtypes, H5 and H7, the arginine residue at the site of cleavage is part of a furin-recognition sequence (8), the presence of which generally correlates with virus pathogenicity (9–11).

The three-dimensional (3D) structures of HA0 and HA, before and after cleavage, differ only near the site of cleavage (12, 13). Nevertheless, cleavage is essential for fusion activity (14, 15). It generates the HA2 N terminus at a conserved hydrophobic sequence, which is called the fusion peptide because its synthetic peptide analogs have membrane fusion activity. It has been envisioned that cleavage and sequestering of the N terminus of the fusion peptide in a conserved pocket primes HA for its response to low pH and that activation of subsequent changes in conformation involves release of the fusion peptide from its buried location (16, 17).

We have previously studied conformational changes in cleaved HA following incubation at fusion pH (18) using cryogenic electron microscopy (cryo-EM) and identified intermediates in the process. To investigate the importance of cleavage to the mechanism of HA-mediated membrane fusion, we now study the response of HA0 to incubation at low pH. We find that the HA0 structure is extensively changed at low pH and that, unlike the changes detected in cleaved HA, the changes in HA0 are reversible on reincubation at neutral pH. We compare the structure of HA0 at low pH with its structure at neutral pH and with one of the low-pH cleaved-HA intermediates (state IV), the “extended intermediate.” This particularly prominent intermediate contains a long central trimeric coiled coil that is assumed to deliver the fusion peptides at its N termini to target cell membranes and to form a bridge between the HA-associated virus membrane and the target. We discuss the possibility that, although not directly involved in membrane fusion, the changes in conformation of HA0 at low pH are indicators of early changes in the conformation of cleaved HA that are required for membrane fusion.

## Results

### HA0 Structure Determined at Neutral pH.

**Fig. 1.**
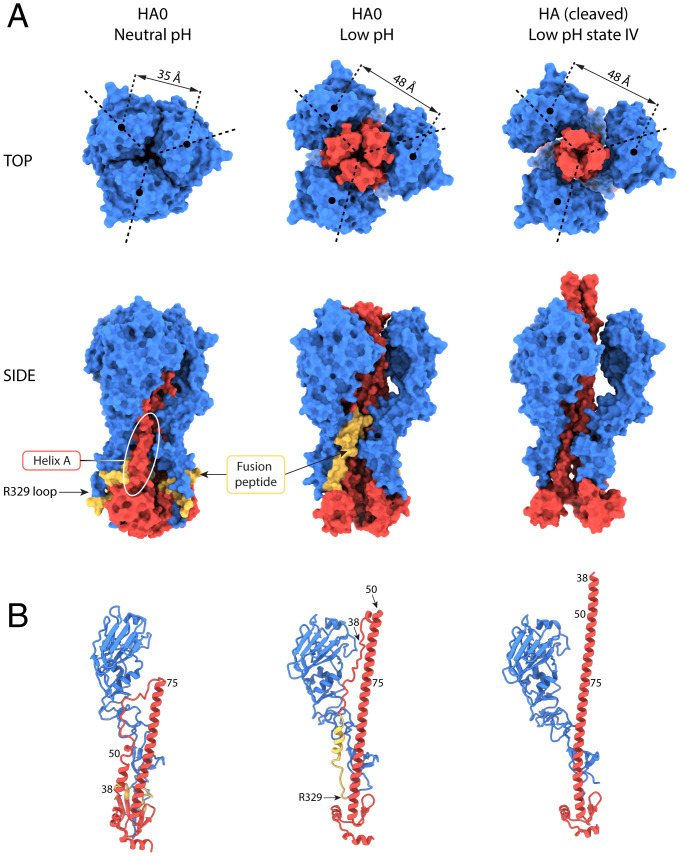
Structural changes at low pH in HA0 and HA. (*A*) Molecular surface representations for HA0 at neutral pH, HA0 at low pH, and HA extended intermediate (state IV, PDB ID: 6Y5K) shown along the trimer axis (*Top* row) and side view (*Bottom* row). HA1 is colored in blue, and HA2 is in red with the fusion peptide (HA2 1 to 23) in yellow. Helix A (HA1 residues 38 to 55) is indicated by the white oval. At low pH, the HA1 membrane-distal domains move outward and the central coiled coil of HA2 extends between them, resulting in a similar conformation to that of the cleaved HA extended intermediate. (*B*) Ribbon diagram of a monomer colored as in *A*.

**Fig. 2.**
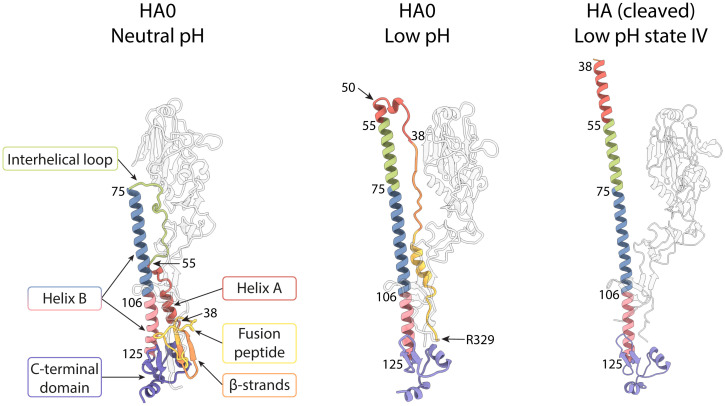
Structural changes at low pH in HA0 and HA highlighting HA2. Ribbon diagram of a monomer showing the conformational rearrangements in HA2 associated with the low-pH–induced transition of HA0 and HA. HA1 is in white; HA2 is colored by structural element: fusion peptide (1 to 23) in yellow, 24 to 37 in orange, A helix (38 to 55) in red, interhelical loop (56 to 75) in green, B helix in blue (76 to 105) and in pink (106 to 125), and C-terminal domain (126 to 172) in purple.

The three-dimensional structure of the X-31 HA0 precursor determined by single-particle cryo-EM at 2.6-Å resolution (Figs. 1 and 2 and *SI Appendix*, Fig. S1 and Table S1) is nearly identical (rmsd 1.3 Å between Cα atoms) to that determined before by X-ray crystallography (12). Two differences are observed. The first one is in the trajectory of the surface loop that contains the site of cleavage into HA1 and HA2 (*SI Appendix*, Fig. S2*A*), which may be influenced by the mutation R329Q introduced at the cleavage site of the HA0 used for X-ray crystallography to prevent adventitious cleavage, or may be a consequence of a crystal contact. The second difference involves loss of a turn and shortening of helix A in HA2 (HA2 residues 38 to 55), and an associated 2-Å shift in the position of the 30 loop (HA1 residues 22 to 37) of the adjacent monomer (*SI Appendix*, Fig. S2*B*). In addition, helix A has a discontinuity at residues 49 and 50 (*SI Appendix*, Figs. S2*B* and S3*A*), with the final helical turn (residues 51 to 54) oriented at 55° relative to the first three turns (residues 38 to 48). This final turn is inserted into a pocket between two of the long central α-helices (B helices) and the 30 loop. Density in the cryo-EM map suggests that this dominant, discontinuous conformation of helix A is in equilibrium (*SI Appendix*, Fig. S3*A*) with the continuous conformation seen in the crystal structure. The two different states are potentially stabilized by different rotamers of His106 in the B helix and Thr30 in the 30 loop (*SI Appendix*, Fig. S4*A*).

The discontinuous conformation of helix A was observed before in crystal structures of HAs from canine and equine influenza viruses (19) and there have been other reports of different HAs with shortened A helices at their C termini (20–22). This region is among those with the highest temperature factors in these and other published HA structures, suggesting that it is conformationally unstable. In the cryo-EM structure, it is also a region of lower resolution.

### The Conformation of HA0 at Low pH.

We have determined the structure of HA0 after incubating it at pH 5.0 to a global resolution of 3.95 Å (Figs. 1 and 2 and *SI Appendix*, Fig. S1 and Table S1). The molecular envelope is wider than that of the neutral-pH structure and extensive rearrangements have occurred throughout the molecule. The membrane-distal domains are dilated and rotated. As in the cleaved HA low-pH extended intermediate (18), dilation of the membrane-distal domains results in a distance of 48 Å between their centroids, compared to 35 Å in the neutral-pH structure (Fig. 1 and *SI Appendix*, Fig. S5) (18). Also associated with dilation, loss of contacts between the membrane-distal domains and the interhelical loop is accompanied by extensive refolding of HA2.

Refolding of the interhelical loop (HA2 residues 55 to 75) and part of helix A leads to extension of helix B from a length of 75 Å to 115 Å (Figs. 1*B* and 2 and *SI Appendix*, Fig. S5). The coiled coil extends between the dilated membrane-distal domains and just beyond them. The extension of helix B in low-pH HA0 (residues 50 to 125) is more limited than that seen in the extended intermediate structure of low-pH cleaved HA (residues 38 to 125), which is about 130 Å in length (Figs. 1*B* and 2 and *SI Appendix*, Fig. S5). The extensions are identical as far as HA2 residue 50, at which point, in low-pH HA0, the coiled coil terminates and is followed by a less ordered, likely helical, turn.

**Fig. 3.**
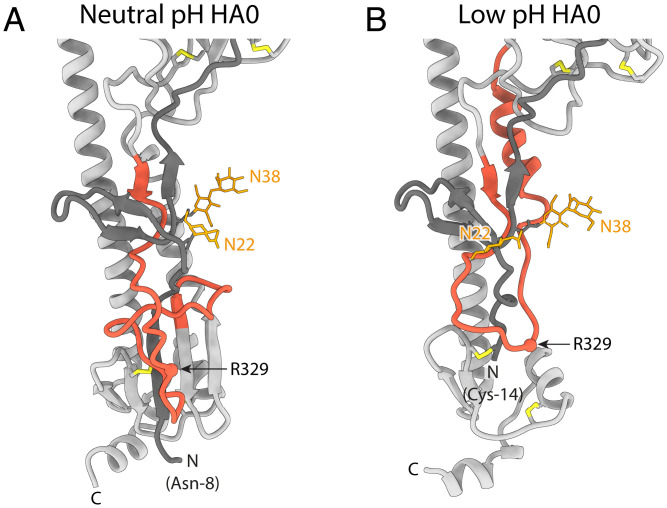
Comparison of the cleavage loop region in HA0 at neutral and low pH. (*A*) Uncleaved HA0 at neutral pH and (*B*) uncleaved HA0 at low pH. Monomers are shown in gray with N-terminal residues of HA1 in a darker gray. The cleavage loop (HA1 315 to HA2 23) is red. Arg329, the site of cleavage, is highlighted as a sphere. Glycans at Asn22 and Asn38 are in orange. In neutral-pH HA0, the cleavage loop is flexible and protrudes from the protein surface into the solvent, while in low-pH HA0 it is constrained to pack around the HA1 N terminus, which is disulfide bonded to HA2 (Cys14 to Cys137).

HA2 residues N-terminal to residue 50 connect the N terminus of the extended helix B to the C terminus of HA1 in the membrane-proximal region. HA2 residues 38 to 44, which form the N terminus of helix A in the neutral-pH structure, adopt an extended chain conformation, running antiparallel to the coiled coil, and are joined by extended HA2 residues 24 to 37, derived from the membrane-proximal five-stranded β-sheet of the neutral-pH structure (Figs. 2 and 3). The fusion peptide (HA2 residues 1 to 23) links this chain to the C terminus of HA1. As shown in Fig. 3, residues forming the solvent-exposed loop of HA0 at neutral pH (HA1 324 to 328, Arg329 and HA2 1 to 7) are constrained at low pH to pack against the surface of the molecule.

The trimeric structure of the membrane-proximal region of HA0 is also opened at low pH and the C-terminal parts of the B helices (HA2 residues 100 to 125) that form a tripod at neutral pH are straightened but to a lesser extent than observed in the low-pH cleaved-HA extended intermediate (Fig. 1 and *SI Appendix*, Fig. S5).

**Fig. 4.**
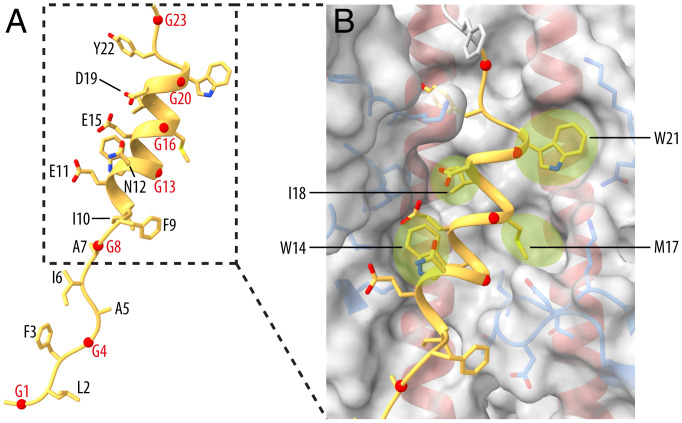
The structure of the fusion peptide in low-pH HA0. (*A*) A ribbon diagram of the fusion peptide (HA2 1 to 23) showing the solvent-exposed face. Glycine residues are labeled in red. (*B*) Closeup view of the three-turn amphipathic α-helical fusion peptide with the molecular surface of the binding site highlighting the pockets (green) for the side chains of fusion peptide residues W14, M17, I18, and W21. Side chains of HA1-contact residues (blue) and HA2 helices (red) are shown.

A striking feature of the structure of low-pH HA0 is the fusion peptide partially folded into an α-helix (Fig. 4 and *SI Appendix*, Fig. S6). HA2 residues 10 to 21 form a three-turn amphipathic α-helix that is positioned at the interface between two B helices and a relocated 30 loop, burying about 65% of its solvent accessible surface area. The polar residues Glu11, Asn12, Glu15, and Asp19 and the glycines Gly8, Gly13, Gly16, and Gly20 are exposed to solvent. The hydrophobic residues Ile10, Trp14, Met17, Ile18, and Trp21 are buried and the last four bind in pockets on either side of the helix. Tyr22 just beyond the helix is also buried as it enters the adjoining channel (Fig. 4). The corresponding hydrophobic binding groove in neutral-pH HA and HA0 structures accommodates the C-terminal region of helix A.

### The Low-pH Conformational Changes in HA0 Are Reversible at Neutral pH.

On returning HA0 to neutral pH after incubation at low pH, we found by cryo-EM reconstruction that it reforms the neutral-pH conformation. This observation was supported by reversible changes in near ultraviolet circular dichroism (UV CD) spectra and by reversible changes in sensitivity to trypsin proteolysis (*SI Appendix*, Fig. S7) (23–25). The increase in intensity of the near UV CD signal of HA0 at pH 5 was reminiscent of that observed before for cleaved HA at pH 5 (25). Reversal to pH 7 restored the signal almost completely, unlike observations made with cleaved HA, which indicated irreversibility (24). Low-pH–specific sensitivity to digestion with trypsin was measured using HA0 or the uncleavable mutant R329Q HA0, so that the changes could be distinguished from those that would occur at low pH if HA0 was cleaved into HA1 and HA2 (24). Both HA0 and R329Q HA0 became sensitive to trypsin below pH 5.5 but, on reversal to pH 7 after 5 min at pH 5, the R329Q mutant HA0 was completely resistant to digestion and HA0 was only cleaved at Arg329 into HA1 and HA2 (*SI Appendix*, Fig. S7 *B* and *C*).

Furthermore, when observed by negative stain electron microscopy, trypsin treatment of low-pH HA0 resulted in the formation of rosettes such as those formed by cleaved HA at membrane fusion pH (*SI Appendix*, Fig. S8). This suggests that low-pH HA0 can undergo the full conformational change once the covalent restriction is removed and that the conformation adopted by HA0 at low pH is on the same refolding pathway as cleaved HA at low pH.

The 3.1-Å reneutralized HA0 structure (*SI Appendix*, Fig. S1 and Table S1) is very similar to that of neutral-pH HA0 (rmsd 0.5 Å between Cα atoms) except that helix A in reneutralized HA0 is a five-turn continuous helix rather than the discontinuous helix A of the neutral-pH HA0 cryo-EM structure. In addition, the 30 loop is relocated about 2 Å higher as in other structures with continuous A helices (*SI Appendix*, Figs. S3*B* and S4*C*). In reneutralized HA0, the hydrophobic groove occupied by residues 16 to 21 of the helical fusion peptide in the low-pH HA0 structure, and by residues 54 to 55 of the discontinuous helix in the neutral-pH HA0 structure, contains density consistent with a molecule of octyl-β-glucoside (*SI Appendix*, Fig. S3*B*). The antiinfluenza virus compounds tert-butyl hydroquinone (TBHQ) (26) and Arbidol (27) have also been shown to bind in this location (*SI Appendix*, Fig. S9).

Reversion to the neutral-pH structure of HA0, therefore, involves refolding of the extension to helix B to form the interhelical loop and helix A; repositioning of the membrane-distal domain, restoration of its intrasubunit and intratrimer interactions and those with the reformed interhelical loop; repositioning of the 30-loop, helix A and the C-terminal region of helix B; reconstruction of the membrane-proximal five-stranded β-sheet by incorporation of the two β-strands linked to helix A; and relocation of the fusion peptide and the cleavage loop.

**Fig. 5.**
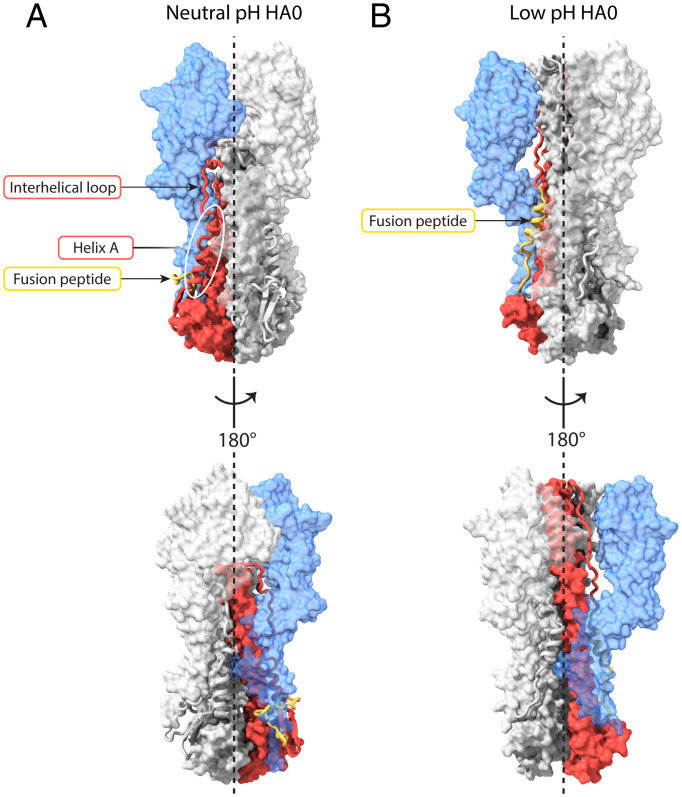
Extension of the coiled coil by threading. Surface and ribbon representations of HA0 showing side view (*Top*), and 180° rotated side view (*Bottom*). One monomer is colored with HA1 in blue and HA2 in red with the fusion peptide (HA2 1 to 23) in yellow. (*A*) For HA0 at neutral pH, helix A (HA1 residues 38 to 55), indicated by the white oval, is linked to the N terminus of the central coiled coil (HA2 residue 75) by the interhelical loop, which traverses a narrow channel between the membrane-distal domain and the central α-helical coiled coil. (*B*) In HA0 at low pH, the fusion peptide (yellow) is linked to the N terminus of the partly extended coiled coil (HA2 residue 50) by HA2 residues 24 to 49, which again traverse through the narrow channel between the now dilated membrane-distal domains and the central α-helical coiled coil. See also Movie S1.

In both HA and HA0 at neutral pH, the interhelical loop connecting the A helix to the N terminus of the coiled coil traverses a narrow cavity between the membrane-distal domains and the central coiled coil (Fig. 5*A* and Movie S1). In HA0 at low pH, the residues N terminal to the central helical extension traverse the same cavity (Fig. 5*B*) following transport of the interhelical loop and part of helix A to the N terminus of the coiled coil. At its narrowest, the available channel has a diameter of about 8 Å by comparison with that of an α-helix of 12 Å. Sequential melting and threading of unfolded structures between the membrane-distal domains and the extending B helices of the central coiled coil is a plausible mechanism for their transport, because the extent of dilation of the membrane-distal domains restricts their transfer en bloc. Threading through this cavity is also the most direct path to reach the top of helix B of the same monomer, which, due to the twist of the growing coiled coil, is on the opposite side of the trimer axis. Formation of the extension to the coiled coil is probably the driving force. On reversal to the neutral-pH structure, the extension of the long central helices melts and threads through the cavity in a reverse direction, likely driven by the folding of neutral-pH structures proximal to the membrane, including the five-stranded β-sheet, facilitated by the covalent linkages between HA1 and HA2.

## Discussion

The extensive structural responses of HA0 to low pH have clear similarities to those seen in cleaved HA (Fig. 1). Prominent among them are the refolded interhelical loop that forms part of the central helical coiled-coil extension, the dilated and rotated membrane-distal domains, and separation of the C-terminal membrane-proximal domains. Dilation and rotation of the membrane-distal domain leads to loss of interactions with the interhelical loop, releasing the loop for changes in its structure at low pH. Sequential addition of residues from the loop and helix A to the N terminus of helix B results in an extension of the central coiled coil in a process that appears to require threading of melted helix A through the restricted space between the dilated membrane-distal domains and the extending B helices. The same considerations are likely to apply to the refolding of the interhelical loop, helix A, and the fusion peptide in cleaved HA at fusion pH. Consistent with these observations, the results of hydrogen–deuterium exchange experiments with cleaved HA suggest that helix A and the interhelical loop sample different secondary structures and may be relatively unstructured during HA activation at low pH (28). On the basis of the similarity of the extended intermediates of cleaved HA and HA0 that are formed at low pH and of the proposal that the extensions of the central helices in HA0 and cleaved HA are likely to involve a common threading path and mechanism, we have interpreted the structure of low-pH HA0 as a captured intermediate that might also be formed during the conformational change in cleaved HA at fusion pH.

In this process, it has generally been considered that priming of HA for its activation at fusion pH occurs as a result of the changes in structure that follow cleavage of HA0 (12, 15). Priming involves sequestration of the N terminus of the highly conserved, hydrophobic fusion peptide into a cavity of ionizable residues with which it interacts and results in an increase in the stability of HA (29, 30). Loss of these interactions at low pH and exposure of the fusion peptide were proposed to be involved in the activation of the conformational changes required for membrane fusion in cleaved HA (15, 16). The finding that the release of the fusion peptide is rate limiting for membrane fusion (31) is consistent with these proposals. Obviously, loss of these specific interactions is not involved in the changes in conformation of uncleaved HA0 at low pH because the fusion peptide is only buried in this cavity following cleavage. Two implications of this conclusion are, firstly, that conformational changes associated with activation, in different regions of HA0 in response to low pH, can occur independently of either priming or activation that result from insertion of the fusion peptide into the cavity or its extrusion from the cavity. Secondly, that the mechanism of HA activation for fusion involves a wider molecular distribution of effects of low pH on interactions established during HA0 folding and subsequent intrasubunit and intersubunit contact. Both implications are consistent with the locations of amino acid substitutions throughout the length of HA that influence the pH of the conformational change (32–34).

What restricts the changes in low-pH HA0 to those we have observed? The covalent linkage of HA1 through Arg329 to HA2 and the conserved disulphide bond between HA1 Cys14 and HA2 Cys137 in the membrane-proximal five-stranded β-structure both contribute to the structural stability of HA0. The former in particular, coupled with the propensity of the components of HA2 to adopt specific structures and interactions, such as the amphipathic helix formed by the fusion peptide, may simply restrict the overall length of the refolded chain. Removal of this restriction may allow the conformational changes observed for cleaved HA at low pH to occur. We have observed that tryptic cleavage of low-pH HA0 without reversal to neutral pH results in the formation of low-pH HA rosettes, i.e., aggregates of soluble HAs formed through interactions of their exposed fusion peptides that are known to be formed by cleaved HA at low pH (23, 24). On this basis, we propose that the changes in conformation of low-pH HA0 occur on a common pathway to that taken by cleaved HA at fusion pH.

A major difference between the responses to low pH of cleaved HA and HA0 is that the changes in cleaved HA appear to be irreversible at neutral pH, whereas the changes in HA0 are reversible. In general, the structure of HA0 has previously been found not to change at low pH, but there have also been reports of reversible and of irreversible changes in different studies (29, 30, 35–38). In some experiments in which monoclonal antibodies were used to assess conformational change at low pH there was no detectable change (35), but in others specific irreversible changes (37) or temperature-dependent irreversible changes were detected, with HA0 changing conformation at higher pH than cleaved HA (29). Reversibility was also suggested in the context of atomic force microscopy imaging experiments (36) and in experiments involving fluorescence resonance energy transfer (FRET) measurements, reversible fluctuations in HA0 structure were also detected, but stable modified structures, as seen for cleaved HA at low pH, were not observed (38). In other experiments, incubation of HA0 in urea, at concentrations lower than those required to change the conformation of cleaved HA, or heating HA0, at temperatures lower than for cleaved HA (30), resulted in changes with some similarities to the structures of cleaved HA, as judged by changes in EM images, antigenicity, and susceptibility to proteolysis. Furthermore, for HA0, some of the data that were interpreted as lack of sensitivity to low pH might have resulted from reversion to neutral-pH conformations before analysis of low-pH–dependent changes (30, 35).

By contrast, the irreversible nature of the overall change in cleaved HA at fusion pH may result from association of the exposed hydrophobic fusion peptides with target membranes or from their formation of protein–protein micelles, such as the rosettes shown in *SI Appendix*, Fig. S8. It may also result from the formation of the N cap that terminates the N-terminal region of the helix-B coiled coil and links it with antiparallel C-terminal residues to colocate the fusion peptide and the membrane anchor regions and to add stability to the rod-shaped molecule formed (39). Nevertheless, the similarity of structures adopted at low pH by HA0 and the extended intermediate of cleaved HA suggests that some of the changes observed in cleaved HA may also be reversible. This may be consistent with reports that the conformation of low-pH cleaved HA is partially reversible under certain conditions of time and or temperature of incubation at low pH, as judged, for example, by changes in CD (40), infrared (41), and fluorescence (42) spectroscopy, by differences in EM images (43), by changes in antigenic properties (44), or by changes in sensitivity to proteolytic digestion (44). Details of the molecular structures formed are not available from these studies, except in the case of a mutant HA for which pH-dependent, reversible changes in the location and orientation of the interhelical loop and deformation of the membrane-distal domains were detailed by X-ray crystallography (45).

The structures formed by HA2 residues 1 to 50 of low-pH HA0 have not been seen in studies of low-pH cleaved HA, we presume because of their transient nature. They suggest that helix A unfolds and refolds in the process of extending the central coiled coil. The two N-terminal β-strands (HA2 24 to 30 and 31 to 37) also unfold and follow helix A and may subsequently contribute flexible structures between the extended coiled coil and fusion peptide that may be required for membrane fusion, as suggested from X-ray crystallography and cryo-EM observations for flexible structures near the HA membrane anchor (46, 47). Additionally, the formation of a helix by residues 10 to 21 of the fusion peptide suggests that, as a component of cleaved HA, the complete fusion peptide, residues 1 to 23, could form a continuous helix, at least transiently, at fusion pH. If these structures are formed in the initial stages of the conformational change in cleaved HA at low pH, they could be considered as targets for potential inhibitors of HA-mediated membrane fusion, as discussed before for intermediates formed by cleaved HA (12, 17, 48). In relation to this possibility, the antivirals TBHQ (26) and Arbidol (27) have both been shown to bind in the hydrophobic groove highlighted here by its proximity to the discontinuity in helix A of HA0 and as the site of insertion of the helical fusion peptide of low-pH HA0. The mechanism of action of these compounds has been proposed to involve an increase in the stability of the neutral-pH structure of cleaved HA (26, 27, 49), but preventing access of the helical fusion peptide to the hydrophobic groove is also a potential mechanism of blocking infection. Similarly, anti-HA monoclonal antibodies, bound in the same hydrophobic groove, could potentially inhibit virus replication. In this connection also, restriction of the movement of the β-strands connected to helix A that are included in the binding sites of other monoclonal antibodies (46, 50) is also a potential mechanism of their antiviral activity.

Synthesis of precursor HA0 as a single chain containing receptor binding, vestigial esterase, and fusion domains facilitates folding of the HA2 region into its neutral-pH conformation. By comparison, expression of HA2 constructs in isolation results in their production in the low-pH conformation (51, 52). Following synthesis in the endoplasmic reticulum, HA0 is transferred to the cell surface through the Golgi network, the pH of which gradually decreases from the cis-Golgi through the trans-Golgi, to the low-pH vesicles that deliver HA0 to the plasma membrane (53). Reversibility of any HA0 structural changes occurring in these low-pH compartments may be required to ensure the production of neutral-pH HA0 for cleavage into HA1 and HA2, which is necessary for the production of infectious virus. If reversibility is required, this would be similar to the situation in the biosynthesis of rhabdovirus fusion glycoproteins, that do not require cleavage for their membrane fusion activity in virus entry but are activated reversibly at low pH (54, 55). Perhaps the precursors of fusion glycoproteins of viruses such as arenaviruses, that require cleavage for fusion activity and are activated in endosomes (56), may also be required to respond to low pH reversibly. However, during influenza virus infection, coexpression of the virus proton channel, M2, probably increases the pH of the pathway sufficiently to avoid exposure of HA0 to low pH. This action of M2 would be analogous to that in which premature low-pH activation of the intracellularly cleaved HAs of highly pathogenic H5 and H7 avian influenza viruses is prevented by M2-mediated increases in the pH of the Golgi (57). In a similar way, M2 could prevent more extensive, irreversible changes in HA0 structure that were reported to occur above 25 °C at low pH (29), and in mutant HA0 structures in FRET experiments (38), if they are found to occur in influenza infections.

Reversibility in HA0 biosynthesis may be an unrecognized requirement in influenza infections and it is also conceivable that cell-surface and virus HA0 may be subjected to conditions of temperature or pH in which reversibility would be advantageous. This appears to be the case for viruses of waterfowl containing HA0, that are exposed to acidic lake water (58).

Among the different structures that HA has been seen to form that may be required for its role in membrane fusion, those made by the fusion peptide are outstanding for their variety. Before cleavage of the HA0 precursor, the fusion peptide forms the membrane-proximal part of a loop that contains the cleavage site, Arg329 (Figs. 1 and 2, and ref. 12). On cleavage, the newly generated N terminus refolds into a two-turn helical structure that is buried in a cavity of ionizable residues, 30 Å from the virus membrane (13). The helical structure reported here for the low-pH form of HA0, that packs into a hydrophobic groove, is the third distinct structure of the fusion peptide to be identified as a component of the protein. In addition to these observations, synthetic peptide analogs of the fusion peptide have been seen by NMR to form 20-residue, V-shaped, helical structures (59) or 23-residue, tightly associated, helical hairpin-like structures (60, 61). There may, therefore, be still other conformations that this conserved region of HA is required to adopt in the formation of a membrane fusion active molecule, and these currently unknown forms may directly give indications of the role of HA in the mechanism of membrane fusion.

## Materials and Methods

### Protein Expression and Purification.

HA0 was recombinantly expressed in Expi293F human cells (Thermo Fisher Scientific). The wild-type (WT) sequence of the HA0 ectodomain from the A/X-31(H3N2) virus strain was cloned into a pOPING expression vector, which incorporates an N-terminal μ-phosphatase secretion signal peptide and a C-terminal His_6_ tag for protein purification. The sequence of the foldon domain of the T4 bacteriophage fibritin was also attached to the C-terminal end of the HA0 ectodomain to promote trimerization of the protein. Cells were transfected using the commercially available ExpiFectamine 293 Transfection Kit (Thermo Fisher Scientific) and incubated at 37 °C in 5% CO_2_ on an orbital shaker at 125 rpm for 3 d, and then at 30 °C for an additional 3 d. On the 6th day, the cell culture supernatant was recovered and HA0 was purified by nitrilotriacetic acid-bound cobalt (Co-NTA) affinity chromatography (HisTALON Superflow Cartridge, Takara Bio) washed with 25 mM phosphate, pH 7.5, 300 mM NaCl, 5 mM imidazole, and eluted with a 5- to 500-mM imidazole gradient, anion-exchange chromatography (HiTrap Q HP, Cytiva) washed with 25 mM Tris⋅HCl, pH 8.0, 50 mM NaCl, and eluted with a 50-mM to 1-M NaCl gradient, and size-exclusion chromatography (Superdex 200, Cytiva). Eluted fractions containing trimeric HA0 were concentrated to 1 mg/mL. The final protein buffer was 45 mM Hepes, pH 7.5, 150 mM NaCl.

The R329Q HA0 mutant was recombinantly expressed in baculovirus-infected insect cells. The sequence of the X-31 HA0 R329Q mutant was cloned into a pFastBac1 vector. The expression cassette places a polyhedrin signal peptide at the N terminus of the R329Q HA0 mutant, and a Tobacco Etch Virus protease cleavage site, a foldon, and a His tag at its C terminus. The vector was transformed in *Escherichia coli* to generate recombinant bacmids. Purified bacmids were transfected into sf9 cells and, 7 d later, the P0 virus stock was harvested. The stock was amplified twice, and the P2 virus was used to inoculate a 2.5-L culture. After 3 d, the supernatant of the culture was harvested. The R329Q HA0 mutant was purified using HisTALON Superflow cartridges (Takara Bio) and incubated with trypsin at a ratio of 20:1 overnight at room temperature to remove the foldon and the His tag. Trypsin inhibitor was added to stop the reaction and the sample was loaded on a Superdex 200 gel filtration column (Cytiva). Eluted fractions containing trimeric protein were pooled and concentrated for further analysis. The R329Q HA0 mutant containing the foldon was purified using the same procedure as for WT HA0.

### Negative Stain Electron Microscopy.

Two-microliter drops of sample were absorbed to a carbon-coated 400 mesh copper grid (TAAB). After 30 s the grid was floated onto water, sample side in contact with the water for 30 s, then transferred to 1% sodium silicotungstate, pH 7.5, (Agar Scientific) for 30 s. The grid was air dried, viewed, and imaged at a pixel size of 4.3 Å with a Technai Spirit transmission electron microscope (FEI) operated at 120 kV, with an Eagle 4K detector (FEI).

### Cryo-EM Sample Preparation and Data Collection.

Grids for cryo-EM were prepared by applying 4 μL of sample onto R2/2 200 mesh Quantifoil copper grids previously glow discharged in the presence of amyl amine, followed by a 4-s blot and plunge freezing into liquid ethane using a Vitrobot Mark IV. Samples of HA0 at pH 7.5 were prepared by diluting the protein stock to 0.25 mg/mL in phosphate-buffered saline. Low-pH samples were prepared by mixing 0.5 mg/mL HA0 with 0.1 M citrate buffer, pH 4.8, in a 1:1 ratio and supplemented with 0.1% octyl-β-glucoside in order to reduce orientational bias, reaching a final pH of 4.8. This low pH was selected to ensure conformational changes in all HA0 molecules at 4 °C. HA0 was incubated at this low pH at 4 °C for 1 or 5 min before transferring it to the grid, also at 4 °C, and plunge freezing. Samples of reneutralized HA0 were prepared by mixing 1.0 mg/mL HA0 with 0.1 M citrate buffer, pH 4.8, in a 2:1 ratio, reaching a final pH of 4.9. The mixture was incubated at 4 °C for 5 min, after which the pH was raised again to 7.0 by adding the same volume of 0.5 M Tris buffer, pH 7.5, supplemented with octyl-β-glucoside to a final concentration of 0.1% octyl-β-glucoside. The mixture was incubated again at 4 °C for 5 min before transferring it to the grid and plunge freezing.

Data were collected on a Titan Krios electron microscope operating at 300 kV. Micrographs were recorded using a Gatan K2 Summit direct electron detector mounted at the end of a Gatan GIF Quantum energy filter, operating in electron-counting mode. A total dose of 41.15 e^−^/Å^2^ was fractionated into 32 movie frames over an 8-s exposure. Images were collected with a calibrated pixel size of 1.08 Å and a defocus range from −1.5 to −3.3 μm.

### Image Processing.

Whole-frame motion correction and dose weighting was done with MotionCor2 (62), and contrast transfer function (CTF) parameters were estimated using CTFFIND4 (63). CrYOLO (64) was used for particle picking by training models specific for the data on a subset of manually picked micrographs. Subsequent processing steps were mostly carried out in RELION-3.1 (65, 66), but cryoSPARC v2 (67) was also used for the refinement of the low-pH structure. First, picked particles were subjected to two rounds of two-dimensional (2D) classification to select the best particles. Those classes that showed secondary structure were selected and used to generate a 3D initial model in RELION, one for each of the two datasets. The best particles were further selected using two rounds of 3D classification and then submitted to 3D autorefinement, imposing C3 symmetry, and to CTF correction and polishing.

For the pH-7.5 dataset, crYOLO picked 2.4 million particles from 16,809 micrographs. 1.5 million particles remained after 2D classification, and after one 3D classification 439,157 particles were selected, yielding a 2.9-Å resolution map after 3D autorefinement and Bayesian polishing. A second round of 3D classification, followed by 3D autorefinement, CTF refinement, and particle polishing, resulted in a 2.6-Å map coming from 122,826 particles.

For the pH-4.8 structure, two datasets were collected after 1- and 5-min incubations at low pH. The 1-min dataset comprised 15,696 micrographs from which 646,027 particles were picked and yielded a 5.9-Å map from 71,971 particles after 2D and 3D classifications in RELION. The 5-min dataset contained 773,138 particles in 18,281 micrographs and, after 2D and 3D classifications, CTF refinement, particle polishing, and refinement in RELION, a map at 4.05-Å global resolution was produced from 137,000 particles. No differences were observed between the two maps, so the best particles from each dataset were added together and used as an input for a heterogeneous refinement in cryoSPARC. The best-defined class contained 149,409 particles and cryoSPARC’s nonuniform refinement produced an improved map at 3.9-Å global resolution.

For the reneutralized dataset, 1.1 million particles were picked from 12,786 micrographs and 656,530 particles remained after 2D classification. The maps of HA0 at pH 7.5 and at pH 4.8 were used as initial models for a two-class 3D classification, which yielded neutral-pH-like structures only. The best particles were further classified and corrected using CTF refinement and Bayesian polishing in RELION and subsequently submitted to 3D autorefinement, generating a 3.1-Å resolution map from 101,306 particles.

### Model Building and Refinement.

Automatically sharpened maps, using RELION for the pH-7.5 and reneutralized structures and using cryoSPARC for the pH-4.8 structure, were used for model building. Postprocessed, denoised maps using the LAFTER (68) and DeepEMhancer (69) algorithms were also used to aid the process. The low-pH HA0 atomic model (comprising residues 14 to 499) was mostly built from the 3.9-Å cryoSPARC map, but residues 360 to 378 (HA2 31 to 49) were built using the 4.05-Å RELION map. High-resolution density with side-chain information for residues 343 to 353 (HA2 14 to 24) allowed sequence assignment and de novo building of the fragment comprising residues 322 to 378 (HA1 322 to HA2 49), for which there was only density for the backbone otherwise. Between residues 358 and 364 (HA2 29 to 35), experimental density suggests that there are at least two paths that the protein could take, but only one was chosen to be built into the model. In the neutral-pH HA0 and reneutralized HA0 structures (comprising residues 8 to 501), residues 327 to 333 (HA1 327 to HA2 4) in the cleavage loop have been built by fitting into low-resolution density. Previously determined structures of X-31 HA (PDB IDs 6Y5H and 6Y5K for the neutral-pH and the low-pH HA0 structures, respectively) were used as a starting point. Manual adjustment of the models was carried out in Coot (70) and ISOLDE (71), refinement was done using REFMAC5 (72) within the CCP-EM software suite, and model geometry and carbohydrate validation were done using MolProbity (73) and Privateer (74), respectively. Channel diameters were calculated with the software HOLE (75). Figures were made using University of California San Francisco ChimeraX (76).

### Near UV CD Spectroscopy.

Near UV CD experiments were done using a Jasco J-815 spectropolarimeter. The spectra were recorded from 340 nm to 255 nm at 25 °C from 50 scans on average and the CD intensity signal was converted to molar CD extinction coefficient (Δε_M_). Δε was calculated on the basis of mean residue weight. Three types of sample were analyzed in 10-mm microfused silica cuvettes: 1) 450 μL of 10 μM HA0, in 25 mM Tris, pH 8.0, 150 mM NaCl, was used; 2) 225 μL of 20 μM HA0 was mixed with 225 μL of 0.1 M citrate buffer, pH 5.0, in equal proportions and the spectra recorded after a 5-min incubation at 25 °C; 3) 120 μL of 40 μM HA0, in 25 mM Tris, pH 8.0, 150 mM NaCl, was mixed with 120 μL of 0.1 M citrate buffer, pH 5.0. After a 5-min incubation at 25 °C, 225 μL of sample was mixed with 225 μL of 1 M Tris buffer, pH 8.0, and the spectra were recorded. In this way, spectra were obtained for HA0 at 1) pH 8.0, 2) pH 5.0, and 3) pH 8.0 following reneutralization after incubation at pH 5.0.

### Trypsin Digestion Assay.

Three 5-μL aliquots of HA0 and R329Q mutant HA0 at 2 mg/mL in 20 mM Tris, pH 7.4, 150 mM NaCl were 1) incubated at 20 °C for 5 min and then 2 μL 0.1% trypsin was added for 5 min followed by 2 μL 0.2% soybean trypsin inhibitor for 5 min; or 2) as in 1 but, before addition of trypsin, acidified by addition of 0.83 μL of 1 M acetate buffer, pH 5.0, for 5 min and then 2 μL 0.1% trypsin added for 5 min and then 2 μL 0.2% soybean trypsin inhibitor for 5 min; or 3) as in 2 but, before addition of trypsin, neutralized to pH 7.5 by addition of 0.83 μL of 1 M Tris for 5 min followed by the addition of 2 μL 0.1% trypsin for 5 min and then 2 μL 0.2% soybean trypsin inhibitor for an additional 5 min. Electrophoresis was at 200 V for 40 min.

## Supplementary Material

Supplementary File

Supplementary File

## Data Availability

Maps and models have been deposited in the Electron Microscopy Data Bank, https://www.ebi.ac.uk/pdbe/emdb/ (Accession Nos. EMD-14742(77), EMD-14743 (78), and EMD-14744 (79)). Models have been deposited in the Protein Data Bank, https://www.ebi.ac.uk/pdbe/ (PDB ID codes 7ZJ6 (80), 7ZJ7 (81), and 7ZJ8 (82)).
