# Supplementary material for: Reversible structural changes in the influenza hemagglutinin precursor at membrane fusion pH
Source: Proc Natl Acad Sci U S A. 2022 Aug 8;119(33):e2208011119. doi: 10.1073/pnas.2208011119 (PMC9388137; doi:10.1073/pnas.2208011119)
Supplement: Supplementary File [file pnas.2208011119.sapp.pdf]

PNAS

Supplementary Information for

Reversible structural changes in the influenza haemagglutinin precursor at membrane fusion pH

Eva Garcia-Moro[a], Jie Zhang[b], Lesley J. Calder[a], Nick R. Brown[b], Steven J. Gamblin[b], John J. Skehel[b], Peter B. Rosenthal[a]

[a]Structural Biology of Cells and Viruses Laboratory, Francis Crick Institute, NW1 AT London, United Kingdom; [b]Structural Biology of Disease Processes Laboratory, Francis Crick Institute, NW1 AT London, United Kingdom.

Peter B. Rosenthal
Email: peter.rosenthal@crick.ac.uk

John J. Skehel
Email: john.skehel@crick.ac.uk

This PDF file includes:

Figures S1 to S12
Table S1
Legend for Movie S1

Other supplementary materials for this manuscript include the following:

Movie S1

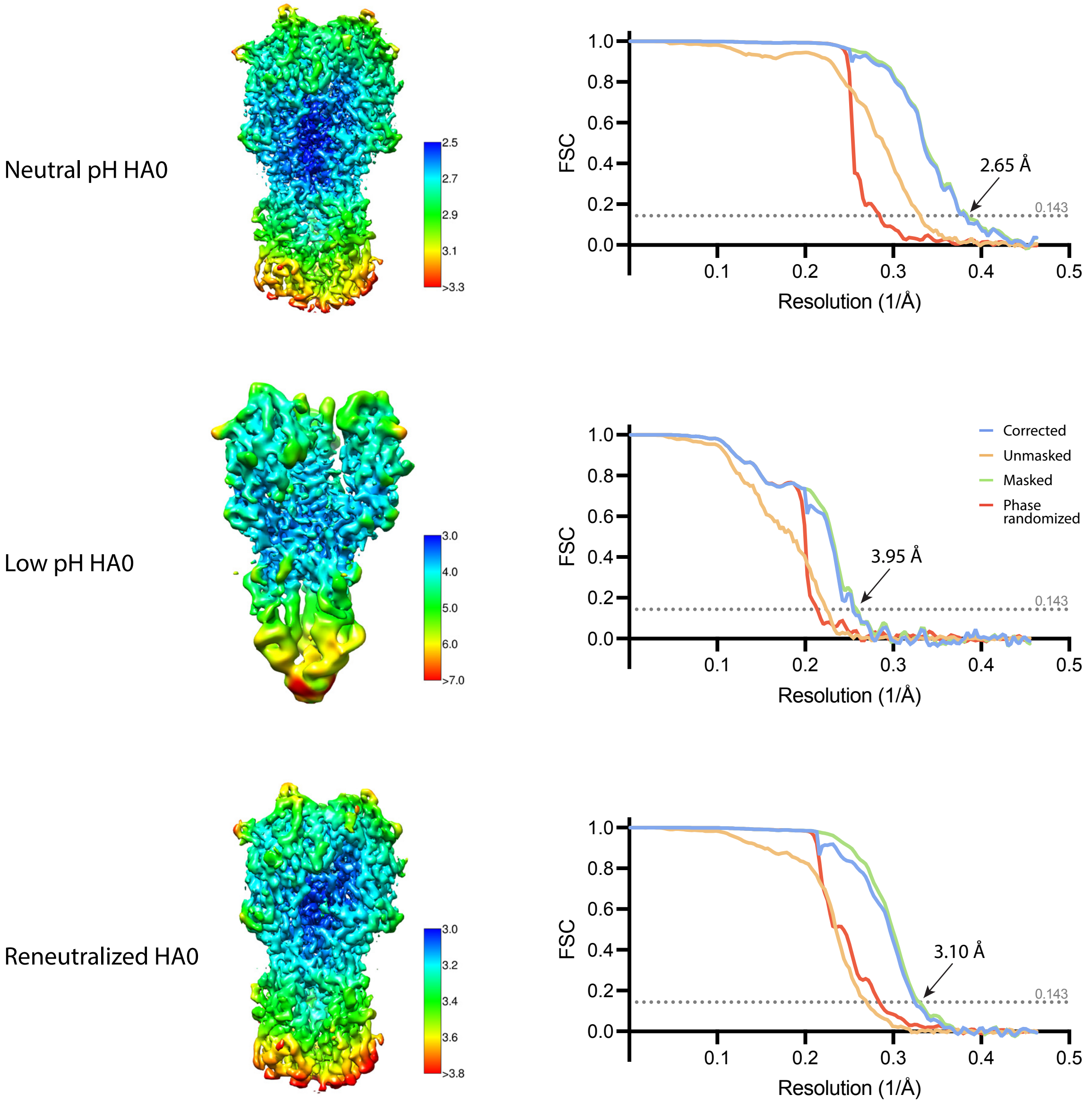

Fig. S1. Local resolution maps and Fourier shell correlation (FSC) curves for all three HA0 cryo-EM structures. Unmasked (orange), masked (green), phase randomized (red), and corrected (blue) curves are shown. Global map resolutions have been estimated using the FSC=0.143 criterion applied to the corrected FSC curve.

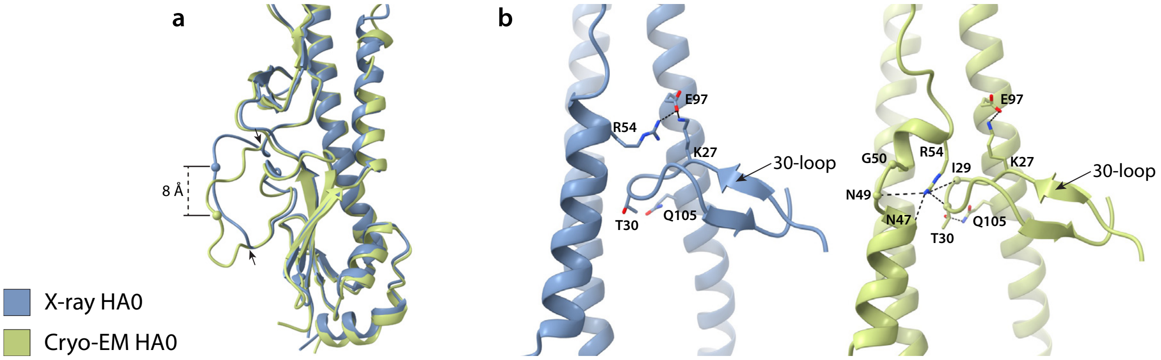

Fig. S2. Differences between HA0 X-ray (PDB ID: 1HA0) and neutral pH cryo-EM structure. (**a**) The trajectory of the cleavage loop diverges between residues HA1 323 and HA2 5 (arrows). Markers indicate the position of Q329 in the X-ray structure and R329 in the cryo-EM structure. In the crystal structure, the 6 residues that precede the cleavage site form part of a circular loop that projects away from the surface. By contrast, in the cryo-EM structure, the residues preceding the cleavage site follow the same path as in cleaved HA and the loop is more elongated. Residues 327 and 328 of HA1, R329, and 1-4 of HA2 are less well resolved than those in the rest of the molecule, probably reflecting flexibility. (**b**) The five-turn short α-helix of HA2 (helix A, residues 38-55) is disrupted in the HA0 cryo-EM structure and the 30-loop is 2 Å lower. The salt bridge between HA2 R54 and HA2 E97 of the neighbouring monomer is lost and R54 points downward, establishing hydrogen bonds with the backbone carbonyls of residues 29 and 30 in the 30-loop, and residues 47 and 49 in the A-helix. Potential hydrogen bonds are represented as black dashed lines.

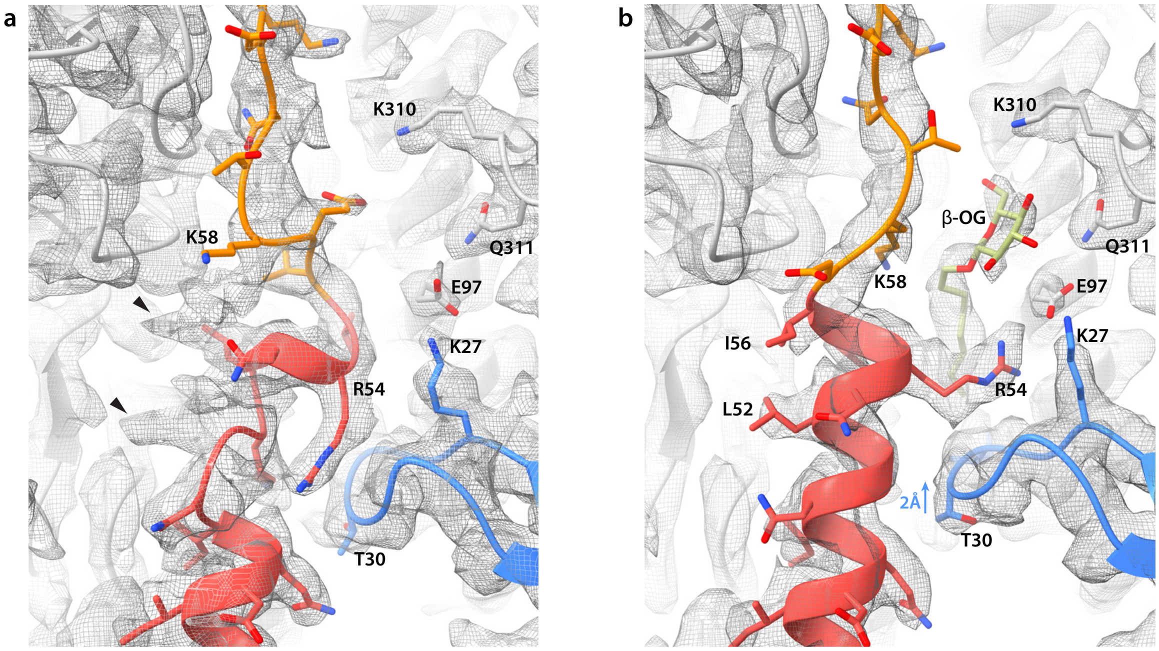

Fig. S3. Helix A in neutral pH HA0 and re-neutralised HA0. In neutral pH HA0 (**a**), density for the discontinuous conformation of helix A predominates, but extra densities where the side chains of L52 and I56 would be expected in the continuous conformation of helix A are also seen (arrow heads). This may indicate that both conformations of helix A are in equilibrium. In re-neutralised HA0 (**b**), helix A adopts the five-turn continuous conformation and the 30-loop is located 2 Å higher. β-OG binds in a hydrophobic pocket adjacent to the C-terminal end of the helix. The aliphatic tail of β-OG is buried among apolar residues, while the pyranose head is exposed to the solvent and likely establishes hydrogen bonds with the nearby charged residue K310. Helix A is in red, interhelical loop is in orange, β-OG is in green, and the 30-loop of the adjacent monomer is in blue. Additional model is in silver. Experimental cryo-EM densities are shown as a meshed surface.

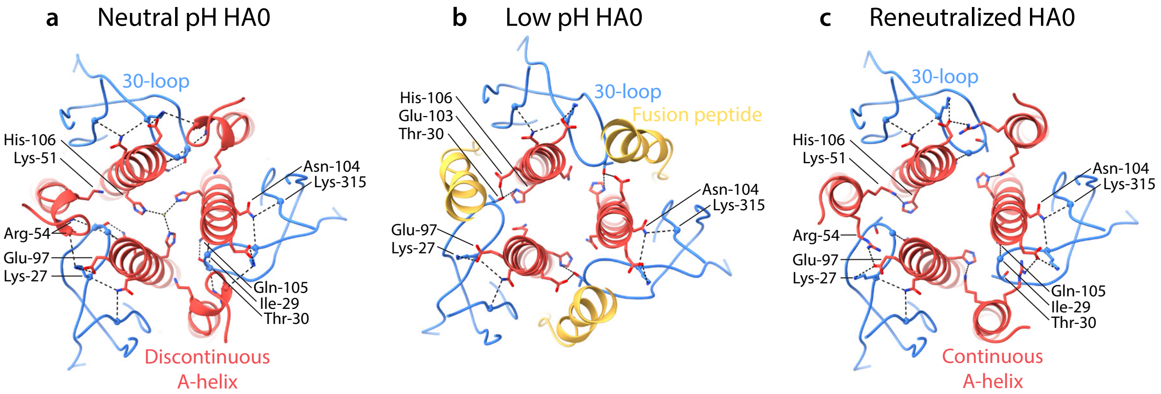

Fig. S4. 30-loop interactions. (**a**) At neutral pH, the 30-loop interacts with R54 in the A-helix and with Q105 in the long α-helix. (**b**) At low pH, the 30-loop rotates slightly with respect to the coiled-coil and T30 potentially establishes hydrogen bonds with E103 and H106 of the neighbouring long α-helix. Helix A is replaced by the helical fusion peptide, which establishes mostly hydrophobic interactions. (**c**) After re-neutralisation from low pH, the 30-loop returns to its original position and interacts with Q105 in the long α-helix, but not with R54 which is now in the five-turn continuous conformation of helix A and forms a salt bridge with E97 in the long α-helix. The salt bridge between K27 in the 30-loop and E97 in the long α-helix is maintained in all structures. Of note, H106 can adopt two rotamer conformations.

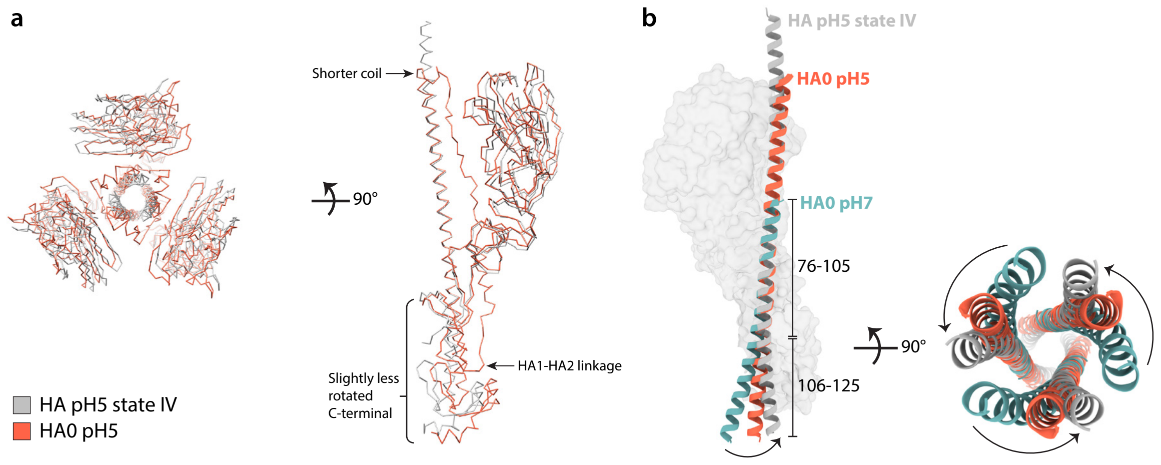

Fig. S5. Comparison of low pH HA0 and HA extended intermediate conformation (state IV). Structures are aligned on the invariant helix of HA2 (residues 76-98). (**a**) Superimposition of low pH HA0 (red) with low pH HA state IV (gray) shown as a top view of the trimer (left) and a side view of a monomer (right). (**b**) Superimposition of HA0 at neutral pH (turquoise), HA0 at low pH (red) and low pH HA state IV (gray). HA1 is depicted as a molecular surface and the long α-helix of HA2 is represented as a ribbon. A side view of a monomer (left) and a bottom-up view of the trimer (right) are shown. Arrows indicate the direction of displacement of the C-terminal part of the long α-helix in the neutral-to-low-pH-transition of HA. In low pH HA0, this straightens to an intermediate position between neutral pH HA0 (indistinguishable from neutral pH HA) and low pH HA state IV.

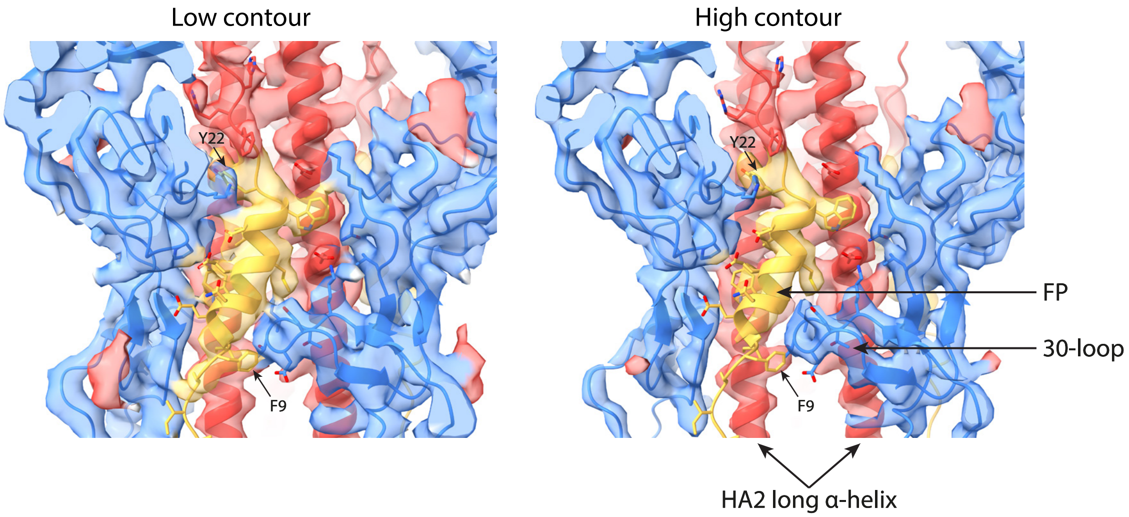

Fig. S6. Cryo-EM density for the fusion peptide and the surrounding area in low pH HA0. The fusion peptide (FP) is in yellow, HA1 is in blue and the remainder of HA2 is in red. Ribbon models with relevant side chains and cryo-EM maps at a lower (left) and higher (right) contour level are shown. Residues F9, W14, M17, I18, W21, Y22 and F24 are identifiable at side chain level in the density.

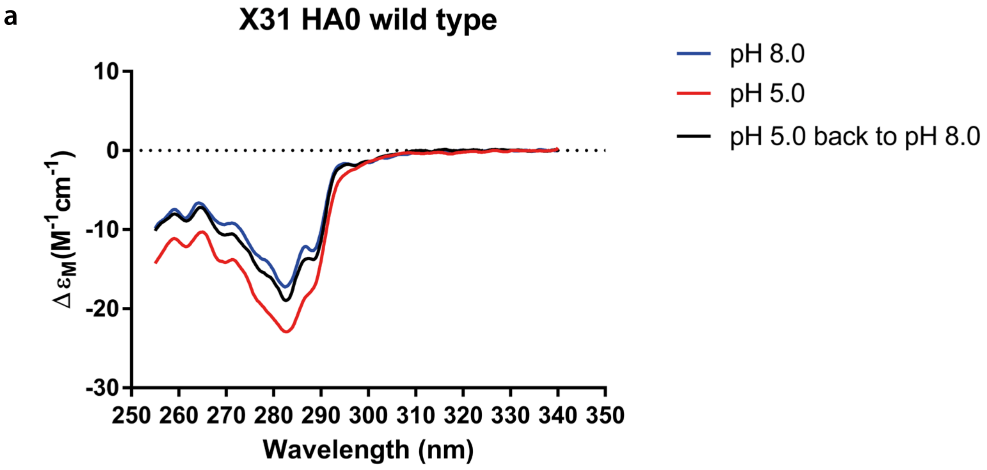

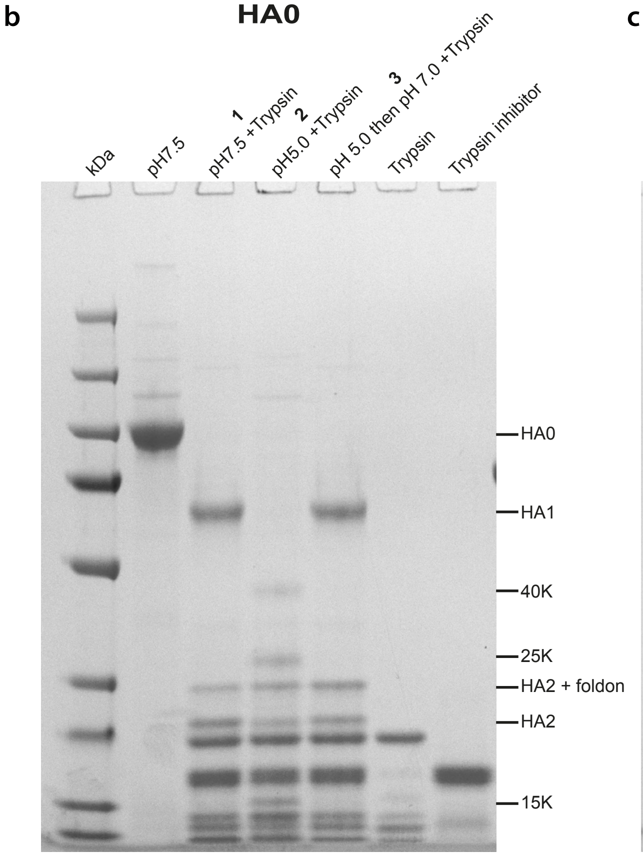

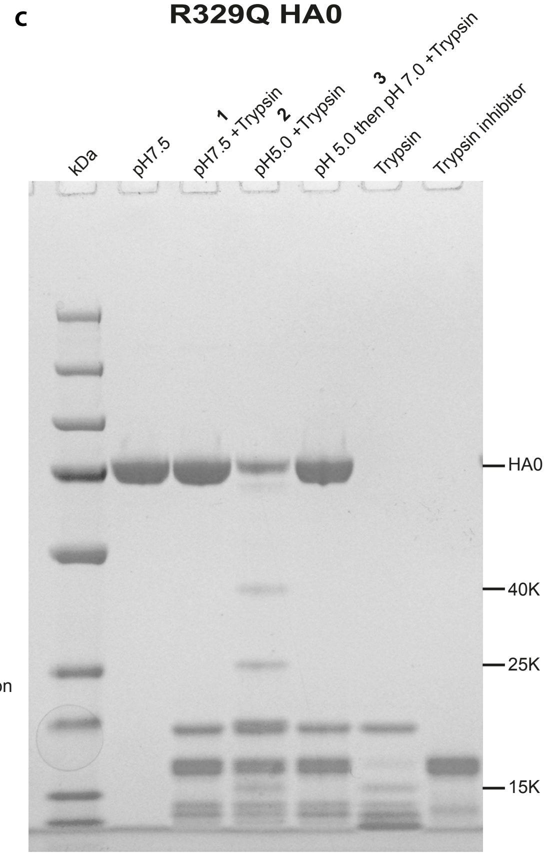

Fig. S7. Near UV CD spectra and susceptibility to trypsin digestion of HA0 as indicators of its conformational change at pH 5.0 and reversal of the change at neutral pH. (**a**) Near UV CD spectra for HA0 were recorded between 255 and 340 nm at 25 °C using a Jasco spectrometer (24) at (1) pH 8.0; (2) pH 5.0; and (3) pH 8.0 following re-neutralisation after incubation at pH 5.0. Gel electrophoresis of (**b**) HA0 or (**c**) R329Q mutant HA0 following trypsin treatment after (1) pH 7.5 incubation for 5 min; (2) pH 5.0 incubation for 5 min; (3) pH 5.0 incubation for 5 min followed by re-neutralisation. As controls, untreated samples of HA0 and R329Q HA0 mutant are included. The products of tryptic digestion are labelled, with 40K, 25K and 15K indicating low pH-specific products of HA1 digestion (24). Additional lanes show trypsin, trypsin inhibitor, and molecular weight markers from 10 to 185 kDa (Thermo Scientific PageRuler Plus).

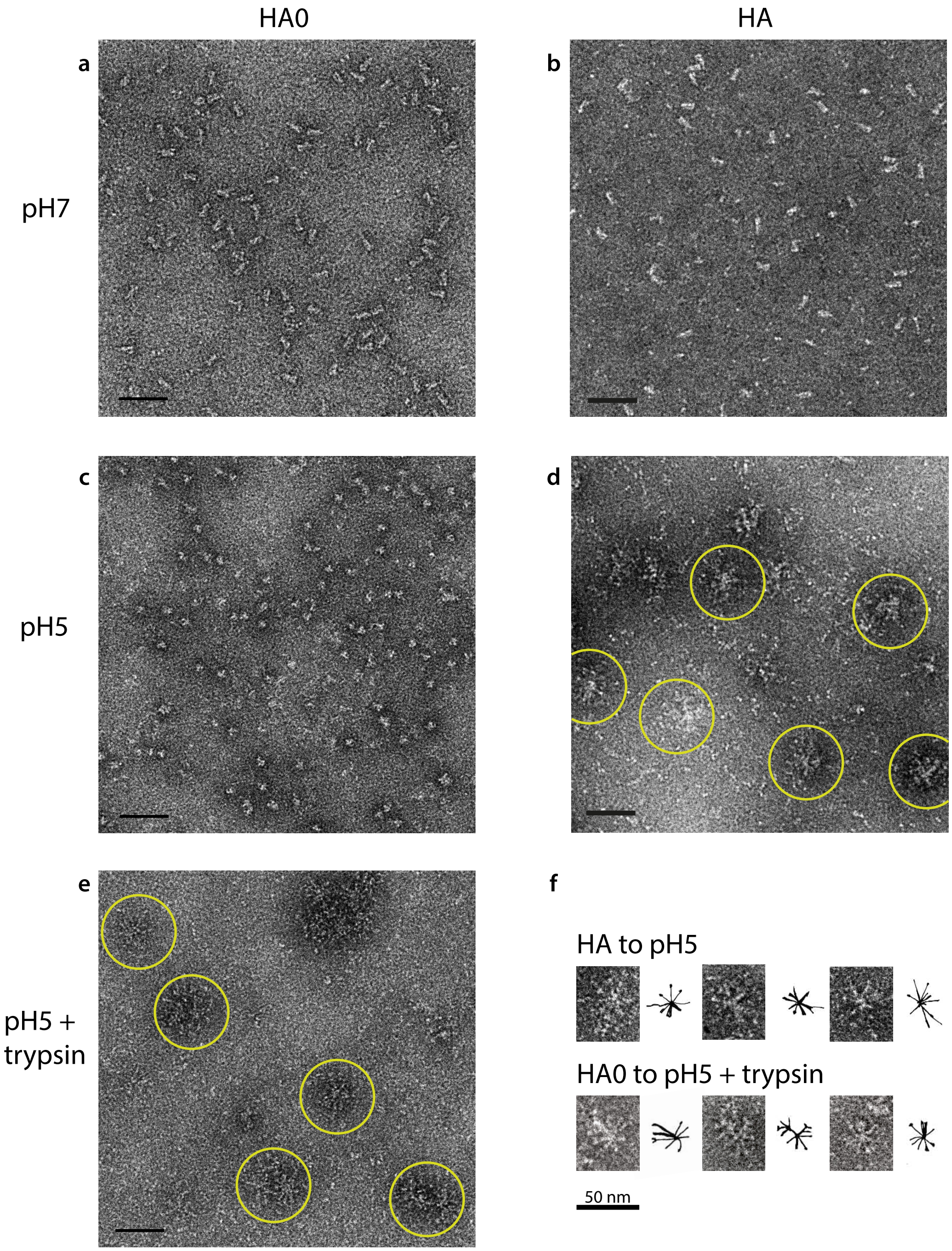

Fig. S8. Structural analysis by negative stain EM of HA0 and cleaved HA at neutral pH and at low pH. The results show (**a**) HA0 incubated at pH 7.0; (**b**) cleaved HA produced by incubation of HA0 with 0.1% trypsin for 4 min at pH 7.0; (**c**) HA0 incubated at pH 5.0 for 5 min; (**d**) cleaved HA incubated at pH 5.0 for 4 min; (**e**) HA0 at pH 5.0 incubated with 0.1% trypsin at pH 5.0 for 4 min. Individual rosettes formed in d) and e) are encircled and (**f**) selected rosettes are shown in the galleries and their structures are illustrated by line drawings. The formation of rosettes such as those in d) results from association of fusion peptides exposed at pH 5.0 in cleaved HA and has been described before (23, 24). The rosettes shown in e) are similar in structure and are proposed to form as a result of exposure of the fusion peptides at pH 5.0 on cleavage of HA0 in the low pH conformation. Their formation is interpreted to indicate that the low pH conformation of HA0 is formed on a similar re-folding pathway to that taken by cleaved HA at pH 5.0.

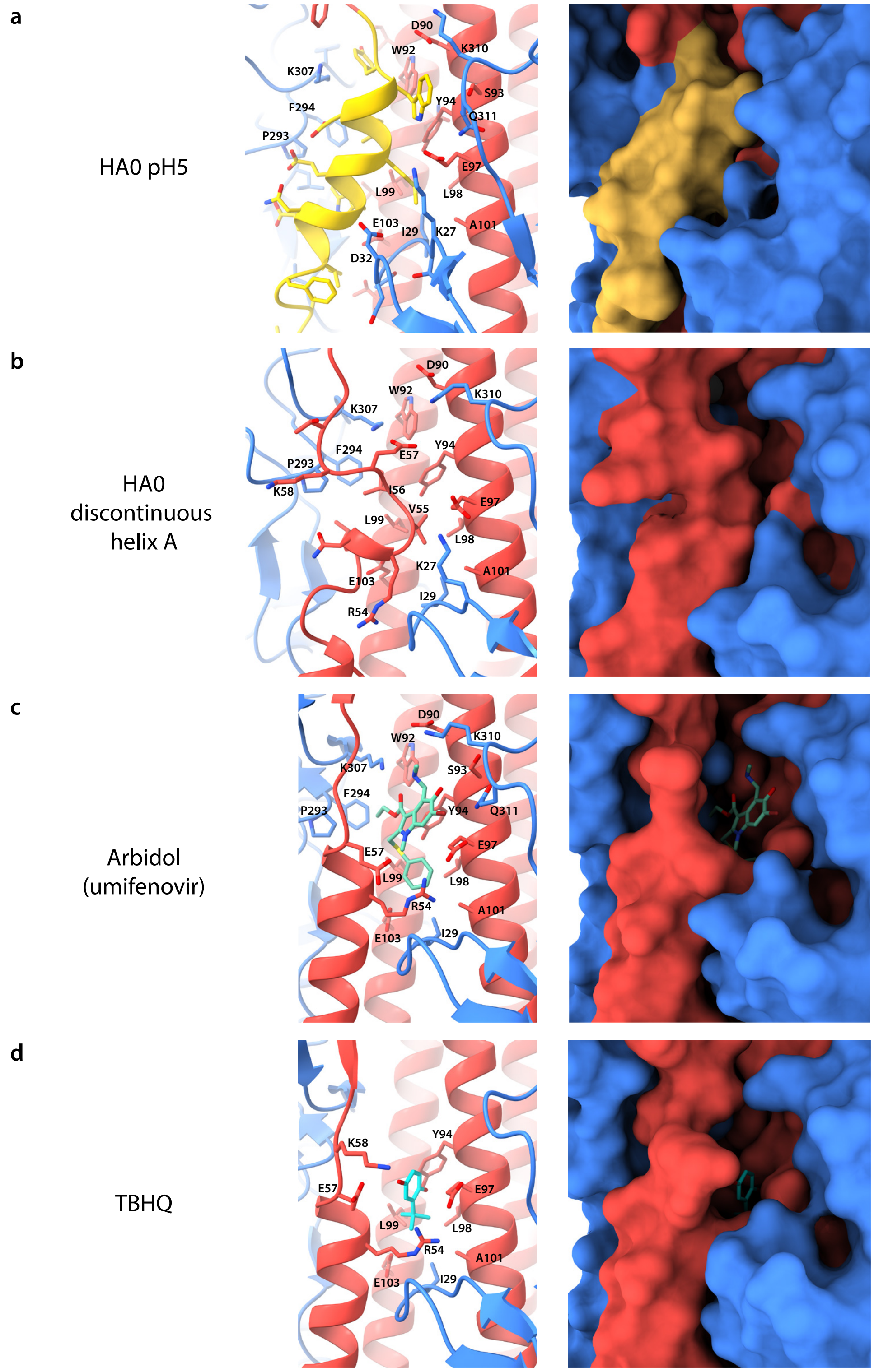

Fig. S9. Location of bound ligands in the hydrophobic binding pocket at the C-terminus of helix A. HA1 is in blue and HA2 in red in all structures. From top to bottom, bound structures are (**a**) the fusion peptide (yellow) in low pH HA0; (**b**) the discontinuous helix A (red) in neutral pH HA0; (**c**) Arbidol (turquoise) in neutral pH HA, from PDB ID: 5T6N; and (**d**) TBHQ (cyan) in neutral pH HA, from PDB ID: 3EYM. Ribbon diagrams (left) show interacting residues, while molecular surfaces (right) show burial of the ligands into the cavity.

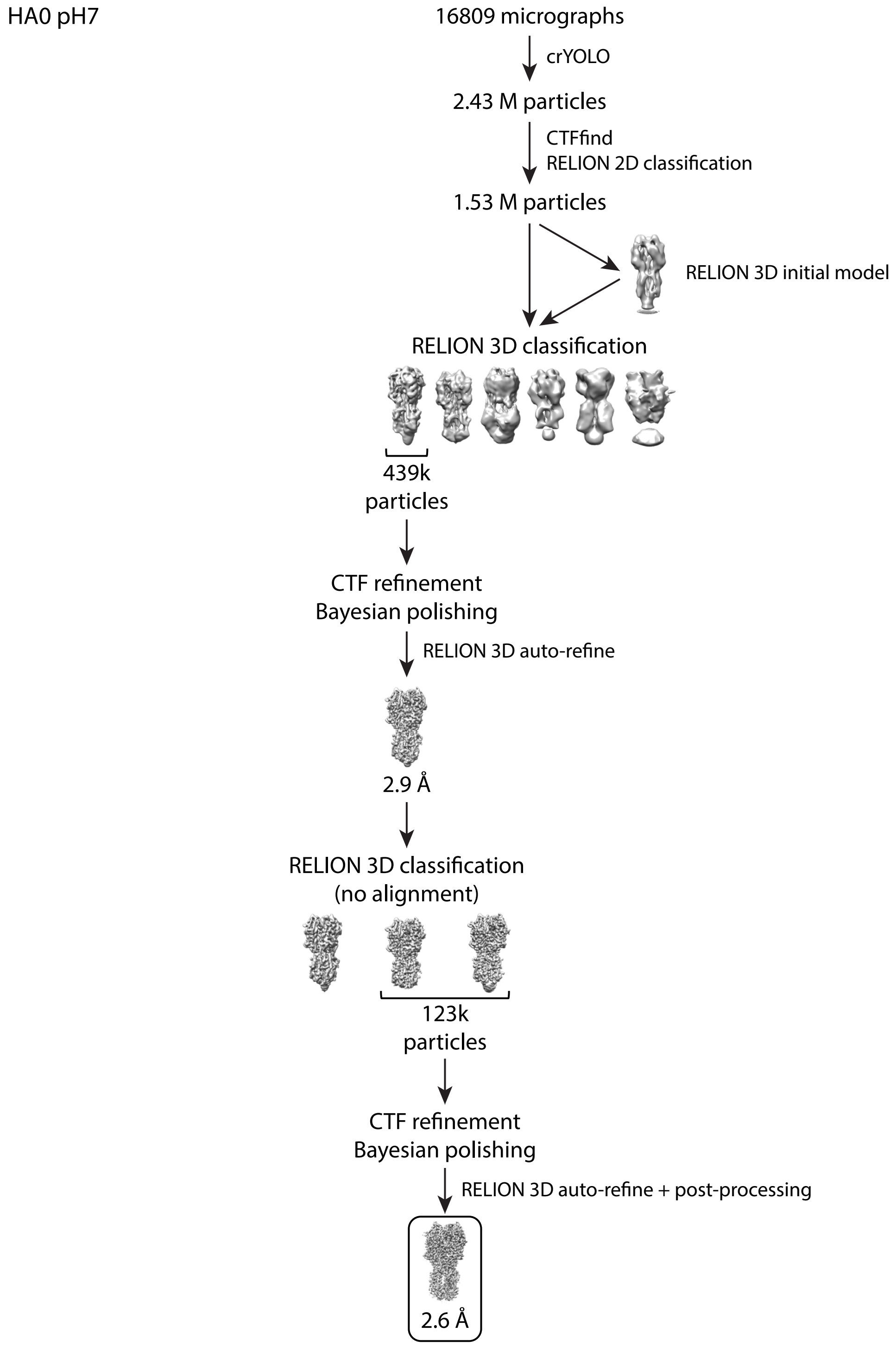

Fig. S10. Cryo-EM image processing workflow for the determination of structures at pH 7.5.

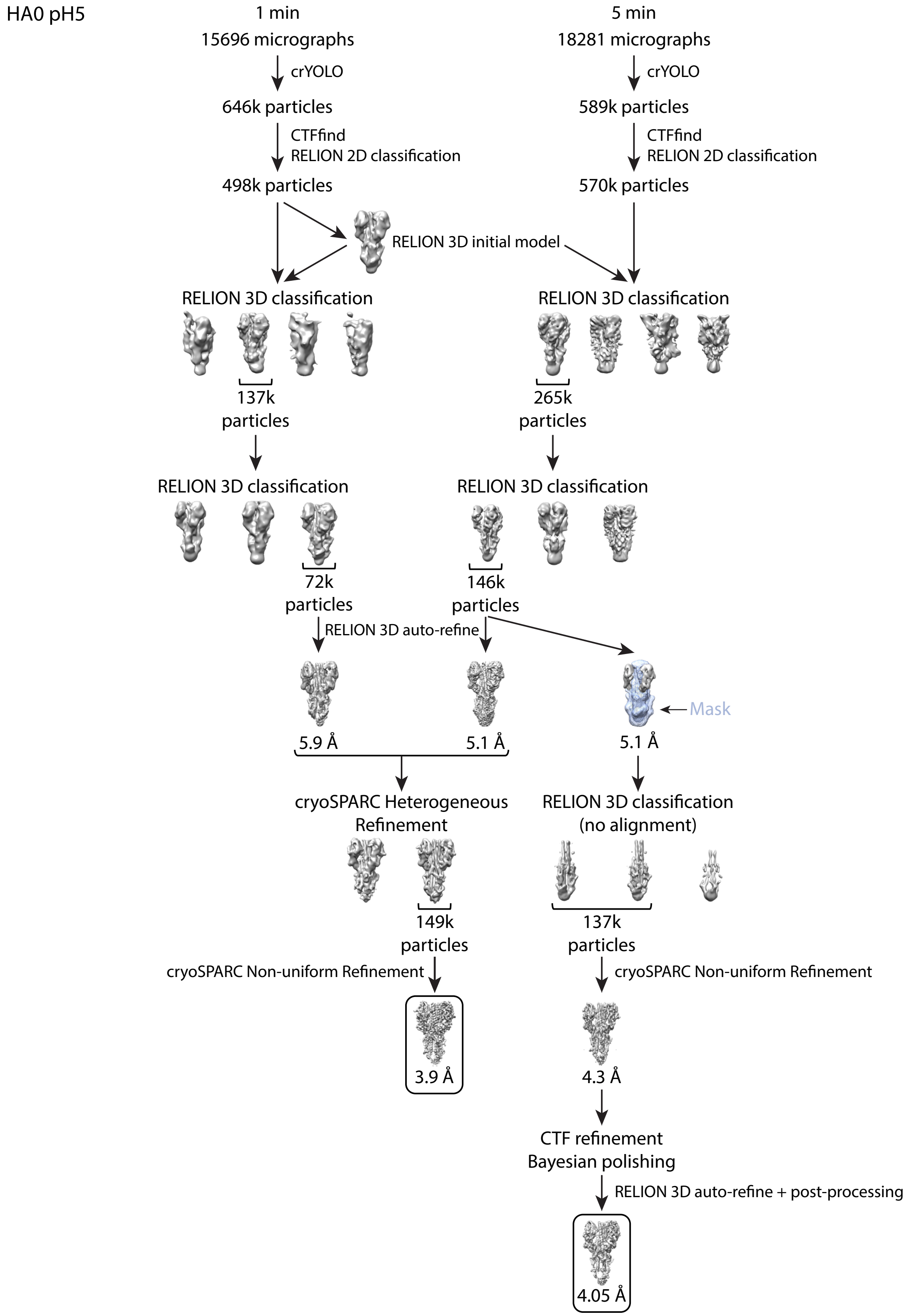

Fig. S11. Cryo-EM image processing workflow for the determination of structures at pH 5.0.

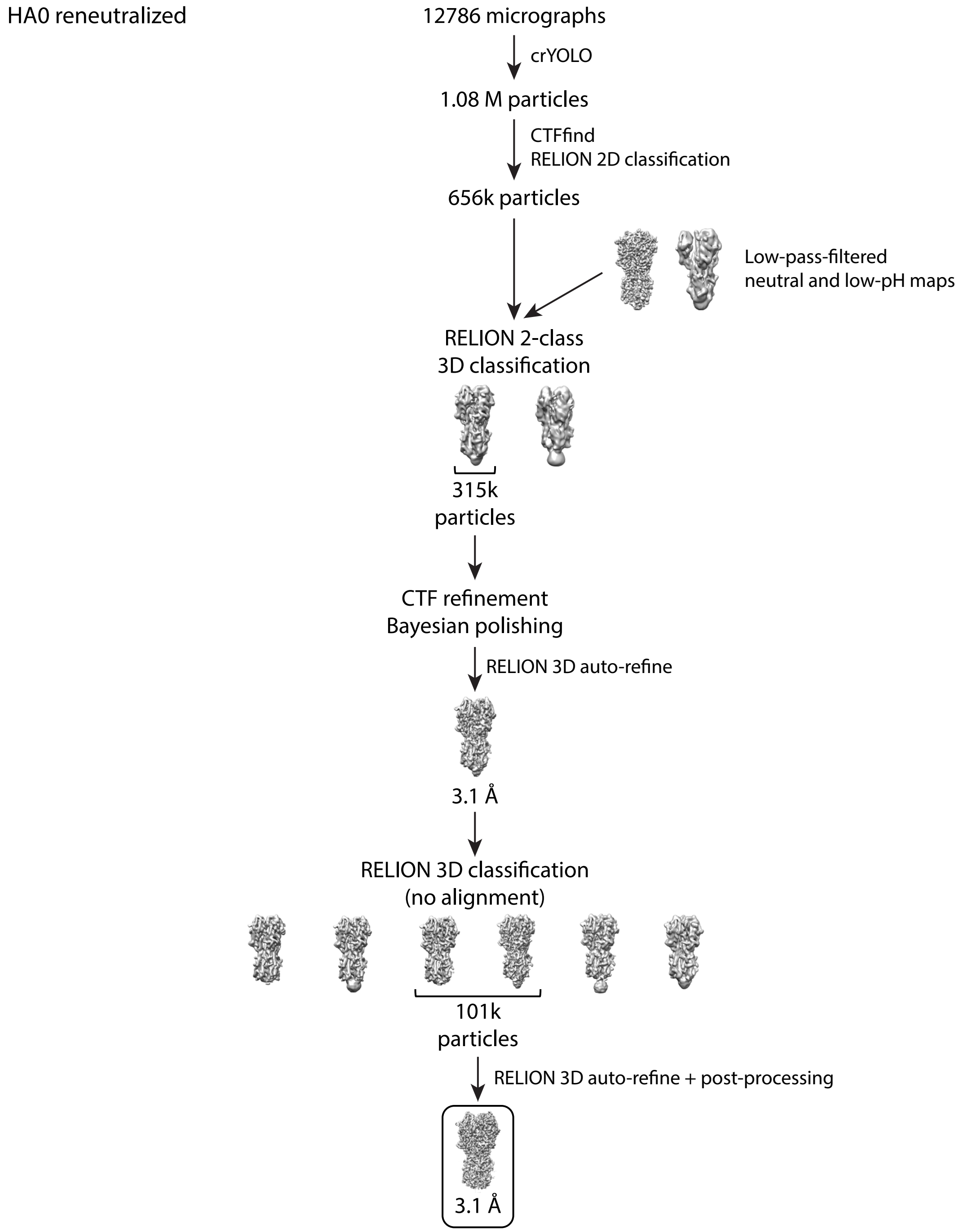

Fig. S12. Cryo-EM image processing workflow for the determination of structures at pH 7.0 after re-neutralization from low pH.

Table S1. Cryo-EM data collection summary and map and model refinement statistics.

	HA0 pH7	HA0 pH5	HA0 reneutralized
**Data collection**			
Microscope	Titan Krios	Titan Krios	Titan Krios
Nominal magnification	130,000	130,000	130,000
Voltage (kV)	300	300	300
Electron exposure (e^-/Å^2)	41.15	41.15	41.15
Defocus range (μm)	-1.5 to -3.3	-1.5 to -3.3	-1.5 to -3.3
Pixel size (Å)	1.08	1.08	1.08
**Processing**			
Micrographs (no.)	16,809	33,977	12,786
Initial particle images (no.)	2.43 M	1.24 M	1.08 M
Final particle images (no.)	123 k	149 k	101 k
Symmetry imposed	C3	C3	C3
Refinement software	RELION	CryoSPARC	RELION
Map resolution (Å) FSC=0.143	2.65	3.95	3.10
Map resolution range (Å)	2.5 to 3.3	3.0 to 7.0	3.0 to 3.8
**Refinement**			
Initial model used	6Y5H	6Y5K	6Y5H
Map sharpening B factor (Å^2)	-60	-180	-90
Map-to-model FSC (Å) FSC=0.5	2.7	4.2	3.2
Model composition			
Non-hydrogen atoms	12282	11958	12342
Protein residues	1482	1458	1482
R.m.s. deviations			
Bond lengths (Å)	0.013	0.013	0.014
Bond angles (°)	1.797	1.886	1.882
Validation			
MolProbity score	1.07	1.28	1.28
Clashscore	1.04	1.87	1.49
Rotamer outliers (%)	0.46	0.55	0.23
Ramachandran plot			
Favored (%)	96.07	95.25	94.11
Allowed (%)	3.93	4.75	5.89
Disallowed (%)	0	0	0

Movie S1 (separate file). Coiled-coil extension by threading. Surface and ribbon representations of HA0 at neutral pH and low pH. One monomer is coloured with HA1 in blue and HA2 in red, with the fusion peptide (HA2 residues 1-23) in yellow.
